# Translation of IRF-1 Restricts Hepatic Interleukin-7 Production to Types I and II Interferons: Implications for Hepatic Immunity

**DOI:** 10.3389/fimmu.2020.581352

**Published:** 2021-01-14

**Authors:** Sabrina Rueschenbaum, Chengcong Cai, Matthias Schmidt, Katharina Schwarzkopf, Ulf Dittmer, Stefan Zeuzem, Christoph Welsch, Christian M. Lange

**Affiliations:** ^1^ Department of Gastroenterology and Hepatology, University Hospital Essen, University of Duisburg-Essen, Essen, Germany; ^2^ Department of Medicine 1, J.W. Goethe University Hospital, Frankfurt, Germany; ^3^ Institute for Virology, University Hospital Essen, University of Duisburg-Essen, Essen, Germany

**Keywords:** liver immunology, macrophages, cytokines, interferons, inflammation, liver cirrhosis, acute-on-chronic liver failure

## Abstract

Interleukin-7 (IL-7) is an important cytokine with pivotal pro-survival functions in the adaptive immune system. However, the role of IL-7 in innate immunity is not fully understood. In the present study, the impact of hepatic IL-7 on innate immune cells was assessed by functional experiments as well as in patients with different stages of liver cirrhosis or acute-on-chronic liver failure (ACLF). Human hepatocytes and liver sinusoidal endothelial cells secreted IL-7 in response to stimulation with interferons (IFNs) of type I and II, yet not type III. *De novo* translation of interferon-response factor-1 (IRF-1) restricted IL-7 production to stimulation with type I and II IFNs. LPS-primed human macrophages were identified as innate immune target cells responding to IL-7 signaling by inactivation of Glycogen synthase kinase-3 (GSK3). IL-7-mediated GSK3 inactivation augmented LPS-induced secretion of pro-inflammatory cytokines and blunted LPS tolerance of macrophages. The IFN-IRF-1-IL-7 axis was present in liver cirrhosis patients. However, liver cirrhosis patients with or without ACLF had significantly lower concentrations of IL-7 in serum compared to healthy controls, which might contribute to LPS-tolerance in these patients. In conclusion, we propose the presence of an inflammatory cascade where IFNs of type I/II induce hepatocellular IL-7 in an IRF-1-restriced way. Beyond its role in adaptive immune responses, IL-7 appears to augment the response of macrophages to LPS and to ameliorate LPS tolerance, which may improve innate immune responses against invading pathogens.

## Introduction

The liver is a highly specialized immunological organ (1). On the one hand, the liver plays an important role in defending the host from pathogens invading *via* the blood stream or *via* the biliary tract. On the other hand, it is highly important that the liver maintains immune tolerance against the large number of harmless foreign substrates (e.g. food components or drugs), which are continuously metabolized by the hepatic excretion machinery (1). This dichotomic function of the liver demands a tight regulation of hepatic immunity, including an appropriate termination of inflammatory responses (1). The special features of hepatic immunity are further illustrated by the presence of large populations of specialized resident immune cells within the liver, including resident T cell subtypes such as CD8+ T cells, CD4+ T cells, γδ T cells, as well as innate immune cells such as NK cells or Kupffer cells (1).

Chronic activation of the hepatic immune system with the consequence of low-grade systemic inflammation is a hallmark of liver cirrhosis (2). Of note, chronic inflammation in liver cirrhosis is accompanied by certain features of immunosuppression, e.g. macrophages of patients with liver cirrhosis poorly respond to lipopolysaccharide (LPS), a phenomenon called LPS tolerance (2). Liver cirrhosis patients are for that reason at high risk of severe bacterial infections. Infections (and other events causing sterile inflammation) can in turn exacerbate systemic inflammation to very high levels causing organ failures such as kidney or circulation failure (2). The occurrence of organ failures in combination with decompensation of liver cirrhosis is defined as acute-on-chronic liver failure (ACLF), which can be considered as the end stage of liver cirrhosis and is burdened with high short-term mortality (2).

Interleukin-7 (IL-7) is a non-redundant cytokine which is essential for T cells, NK cells and γδ T cells to undergo appropriate differentiation, maturation and survival (3). The best known sources of IL-7 are found within the primary lymphatic tissues, where stromal cells express IL-7 constitutively (3). Still, several recent articles have demonstrated that production of IL-7 can be induced by tumor necrosis factor-(TNF)-α or interferon-(IFN)-γ in several non-lymphatic tissues including the murine liver (4). However, the exact functionalities of IL-7 in peripheral organs are not clear yet.

IFNs are central actors in adaptive as well as innate immune responses. Even though they share functional aspects, IFNs of type I (being IFN-α, -β for the most part) and of type III (being IFN-λ1-3) feature early innate immune responses against multiple viruses, for example hepatitis B virus (HBV) or hepatitis C virus (HCV), where IFNs of type II (only IFN-γ) are thought to build successive adaptive immune responses (5). Of note, type I as well as type III IFNs rely on the JAK-STAT signaling pathway and by that promote the expression of numerous overlapping, antivirally active interferon-stimulated genes (ISGs), even though they bind to their distinct receptors, the IFN-α receptor (IFNAR) complex for type I IFNs, and the heterodimeric IL-28Rα/IL-10R2 receptor complex for type III IFNs (5). Importantly, the IL-28Rα/IL-10R2 receptor complex is only present on hepatocytes, plasmacytoid dendritic cells and epithelial cells, whereas the IFNAR is ubiquitously expressed (6).

In this study we aimed to better characterize regulation as well as functional impact of the hepatic IFN–IL-7 axis and to explore consequences for the hepatic innate immune system, a key driver of liver cirrhosis and ACLF.

## Materials and Methods

### Cell Culture, Co-Culture, Reagents

The human cell lines Huh-7.5 (provided by Charles M. Rice, The Rockefeller University, New York, NY) and HepG2 cells (ATCC) were cultured as described previously (7). Primary human hepatic sinusoidal endothelial cells (HLSEC) were cultured in Endothelial Cell Medium in plates/flasks, which were coated with Bovine Plasma Fibronectin (cells and reagents were from ScienCell Research Laboratories). Human monocyte-derived macrophages (MDMs) were isolated from healthy donor buffy coats and maturated as described previously (8). To generate mature dendritic cells (mDCs), monocytes were cultivated in RPMI with 10% FBS, 1% penicillin-streptomycin, 10 ng/ml rhGM-CSF and 20 ng/ml rhIL-4 (both from Peprotech) for 8 days with additional 10 ng/ml LPS during the last two days of differentiation. Isolation of Naïve CD4+ T cells was performed by using the Naïve CD4+ T Cell Isolation Kit II (Miltenyi Biotec). Human peritoneal macrophages were obtained from ascites fluid by centrifugation, then cells were allowed to adhere to cell culture plates overnight. For co-culture assays, cells were plated into Transwells with 0.4 µm pore size (Sarstedt). Human IFN-α2a was a gift from Roche. Human IFN-α1, IFN-α6, and IFN-α14 were provided by Ulf Dittmer (Universtiy of Duisburg-Essen). Human IFNβ, IFN-γ, and IFN-λ2 and rhIL-7 were purchased from PeproTech. Cycloheximide and CHIR99021 were purchased from Sigma. TLR agonists poly(I:C) HMW and Pam3CSK4 were purchased from Invivogen.

### Quantitative Real-Time PCR

Cellular mRNA extraction, cDNA production and quantitative real-time PCR (SYBR^®^ Green-Technology) were conducted as described previously (7), using the following primers: IL-7-fwd 5′-CCTGATCCTTGTTCTGTTGC-3´ and IL-7-rev 5′-TGATCGATGCTGACCATTAGA-3’; ISG15-fwd 5’-TCCTGCTGGTGGTGGACAA-3’ and ISG15-rev 5’-TTGTTATTCCTCACCAGGATGCT-3’; GBP5-fwd 5’-CGCAAAGGTTGGCGGCGATT-3’ and GBP5-rev 5’- AGCTGTGCAGCCTGTTCCTGC-3’; IRF-1-fwd 5’-CCACTCTGCCTGATGACCAC-3’ and IRF-1-rev 5’-GGTGCTGTCCGGCACAACTT-3’; CD127-fwd 5’-TGGAGACTTGGAAGATGCAG-3’ and CD127-rev 5’-AAGCACAGGTCAGTGAGTGC-3’; CD132-fwd 5’-CTTTTCGGCCTGGAGTGGTG-3’ and CD132-rev 5’-ACGCAGGTGGGTTGAATGAA-3’; IL-1β-fwd 5’-CGAATCTCCGACCACCACTAC-3’ and IL-1β-rev 5’-GCACATAAGCCTCGTTATCCC-3’; IL-6-fwd 5’-GCCATCCACCTCTTCAGAACG-3’ and IL-6-rev 5’-CCGTCGAGGATGTACCGAATT-3’; TNFα-fwd 5’-GGCAGTCAGATCATCTTCTCGAA-3’ and TNFα-rev 5’-GAAGGCCTAAGGTCCACTTGTGT-3’.

### Immunofluorescence

Immunofluorescence of MDMs and human liver sections was performed using primary antibodies for CD127, CD132, IRF-1, GSK3, CD68, and CK18, as well as secondary antibodies Alexa Fluor^®^ 555 goat anti-mouse IgG (A21422, Life Technologies) and Alexa Fluor^®^ 633 goat anti-rabbit IgG (A21071, Life Technologies), and bisbenzimide H 33342 trihydrochloride (Sigma Aldrich) as nuclear staining, as described (9).

### Endotoxin Quantification

Media and reagents were free of endotoxin, as confirmed by the use of the Pierce™ LAL Chromogenic Endotoxin Quantitation Kit (Thermo Fisher).

### Antibodies

Antibodies against IL-7 (ab103618), β-Actin (A1978), Lamin B (sc-365962), and γ-Adaptin (610385) were from Abcam, Sigma, Santa Cruz, and BD Bioscience, respectively. Antibodies against CD127 (MAB306) and CD132 (AF284) were from R&D Systems. Antibodies against phospho-Stat5 (Tyr694, 4322), Stat5 (9363) phospho-GSK3 (Ser21/9, 8566), GSK3 (5676) and IRF-1 (8478) were from Cell Signaling Technologies. Antibodies against CK-18 (M7010) and CD68 (M0876) were from Dako. Neutralizing antibodies against IL-7 (MAB207) and IFNAR1 (AF245) were from R&D Systems. For flow cytometric analysis, BV605 anti-human CD127 (351334) and PerCP/Cy5.5 anti-human CD14 (367110) were purchased together with Zombie Aqua Live/Dead Staining (423102) from BioLegend.

### Immunoblotting

Immunoblotting was performed as described previously (7).

### ELISA

Levels of human IL-7, IFNγ, IL-6, and TNFα in human serum samples as well as cell culture supernatant were analyzed using the Human IL-7 High sensitivity, IFNγ, IL-6, and TNFα Quantikine ELISA Kits (R&D systems), respectively.

### Preparation of Cytosolic and Nuclear Extracts

Cytosolic and nuclear extracts were generated as described previously (7).

### Quantification of ATP and Assessment of Cellular Viability

For measurement of intracellular ATP content cells were stimulated as indicated and lysed in buffer containing 0.05 M Tris-HCl at pH7.4, 0.15 M NaCl, 0.002 M EDTA and 1% Nonidet P-40. Cell lysates were analyzed using an ATP Bioluminescent Assay Kit (Sigma) with an EnVision^®^ 2104 Multilabel Plate Reader (Perkin Elmer). Cellular viability was assessed using the WST-1-reagent as described previously (7).

### Gene Silencing

Gene silencing experiments were conducted as described previously (7) by the usage of pre-designed, commercially available small interfering RNAs (siRNAs): s7501 or s7502 for IRF1 (both Cat# 4392420), or non-targeting control siRNA (Cat# 4390843, all from Applied Biosystems).

### Patient Samples

Consecutive patients admitted to the University Hospital Frankfurt, Germany, being diagnosed with either compensated liver cirrhosis, or decompensated liver cirrhosis or acute-on-chronic liver failure in accordance with the criteria of the CLIF-EASL consortium (10), were prospectively enrolled in the present liver cirrhosis cohort study since August 2013 and until April 2017, as described previously in detail (9). Biomaterials were collected at baseline of admission. The definition of acute decompensation of liver cirrhosis included the occurrence of one of the following criteria: bacterial infection, gastrointestinal hemorrhage, new onset/progression of hepatic encephalopathy graded by West-Haven criteria, or ascites grade II-III. All patients in the study provided written informed consent to the protocol, and the study was approved by the local ethic committee of the University Hospital Frankfurt, Germany.

### Statistical Analyses

Group differences of continuous variables were tested by the usage of the Wilcoxon-Mann-Whitney *U* tests. *P* < 0.05 (two-sided) were considered significant.

## Results

### Hepatic IL-7 Is Induced by Types I and II IFNs, Not by IFN-λ

**Figure 1:**
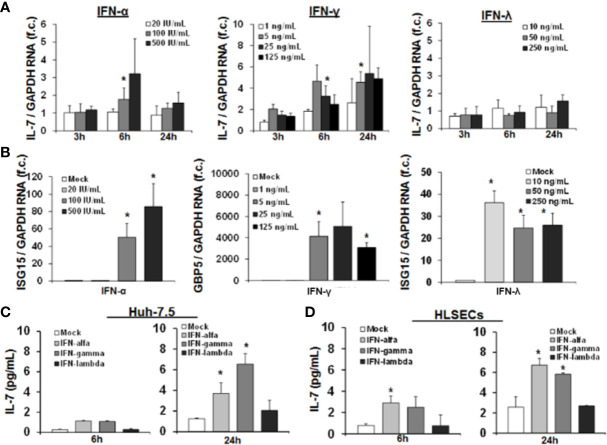
Interferon-(IFN)-α and IFN-γ, but not IFN-λ, induce hepatic interleukin-7 (IL-7) expression and secretion. **(A)** Quantification of IL-7 mRNA levels in relation to housekeeping GAPDH mRNA in Huh-7.5 cells, which were stimulated for the indicated hours with IFN-α (left), IFN-γ (middle), or IFN-λ2 (right) at the indicated dosages. IL-7 expression is shown relative to untreated cells. **(B)** IFN-λ signaling is present in Huh-7.5 cells. Quantification of interferon-stimulated gene (ISG) mRNA levels relative to housekeeping GAPDH mRNA in Huh-7.5 cells, which were stimulated with IFN-α, IFN-γ, or IFN-λ2 at the indicated dosages for 6 h. ISG expression is shown relative to untreated cells. **(C)** Analysis of protein levels of IL- *via* ELISA in cell culture supernatant of Huh7.5 cells and **(D)** primary human hepatic sinusoidal endothelial cell (HLSEC), which were stimulated with IFN-α (500 i.E./ml), IFN-γ (5 ng/ml), or IFN-λ2 (50 ng/ml) for 6 or 24 h. In **(A–D)**, standard deviations of three independent experiments (n=3) performed with three replicates each are shown. *P < 0.05 for comparison of the indicated condition with mock. f.c., fold change. IU, international units.

At first, we first assessed IL-7 induction in the important human Huh-7.5 cell line, in which for example HCV infection can be studied *in vitro*. As shown in **Figure 1A**, IL-7 gene expression was induced by IFN-α in a dose dependent manner with a maximal induction after 6 h of treatment. Furthermore, IFN-γ treatment of Huh-7.5 cells led to a robust induction of IL-7 gene expression. Of note, IL-7 was profoundly induced even by low doses of IFN-γ, and IL-7 induction was longer lasting after stimulation with IFN-γ vs. IFN-α, respectively (**Figure 1A**). A pronounced induction of IL-7 in Huh-7.5 cells, as well as in HepG2 cells, was also observed after stimulation with IFN-β and with some - but notably not all - IFN-α subtypes (**SI Figure 1**). In contrast to treatment with type I and II-IFNs, stimulation of Huh-7.5 cells with IFN-λ2 did not induce IL-7 gene expression (**Figure 1A**), although IFN-α and IFN-λ2 are considered to share the intracellular JAK-STAT signaling cascade components. To exclude dysfunctional IFN-λ signaling in our cell lines, we assessed induction of signature ISGs for type I, II, and III IFNs. In these experiments, stimulation of Huh-7.5 cells with IFN-λ2 resulted in a strong increase of ISG15 expression (**Figure 1B**), indicating that the IFN-λ signaling pathway is intact in our model. Of note, non-specific stimulation of Huh-7.5 cells and HepG2 cells with LPS did not result in induction of IL-7, confirming the specificity of the observed findings (**SI Figure 1**).

Next, we confirmed hepatocellular IL-7 expression on the protein level by quantification of secreted IL-7 in the supernatant of IFN-stimulated Huh-7.5 cells. An increase of IL-7 protein levels in supernatants of Huh-7.5 cells was observed 24 h after treatment with IFN-α or IFN-γ (**Figure 1C**), resulting in concentrations reflecting a physiological range *in vivo* (see **Figure 8**). To further substantiate the possible *in vivo* relevance of these finding, we assessed IL-7 production of primary human hepatic sinusoidal endothelial cells (HLSECs), which represent a key component of the hepatic immune compartment and which are well-known targets of IFN-signaling (11). Stimulation of these primary HLSECs with both type I or II IFN resulted in a significant induction and secretion of IL-7, comparable to that of hepatoma cell lines (**Figure 1D**).

Collectively, these data proof a relevant production of hepatic IL-7 due to signaling of type I and II IFN in the human system.

### Translation of IRF1 Restricts Hepatocellular IL-7-Induction to Types I and II IFN

**Figure 2:**
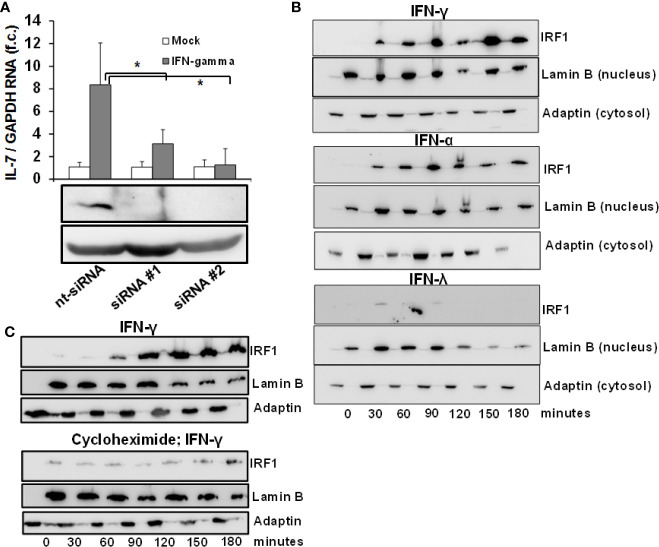
Inducible interleukin-7 (IL-7)-expression in the hepatic system relies on interferon-response factor-1 (IRF1) protein translation. **(A)** Huh7.5 cells underwent gene silencing by treatment with IRF-1 siRNA or unspecific control small interfering RNA (siRNA) for 24 h. After removing the siRNA, cells recovered for 24 h before being treated with 5 ng/ml interferon- (IFN)-γ for 6 h. Levels of IL-7 mRNA relative to housekeeping GAPDH mRNA are expressed relative to untreated cells. Standard deviations of two independent experiments (n=2) performed with three replicates each are shown. *P < 0.05 for comparison of IFN-gamma treatment in nt-siRNA vs. siRNA#1 or siRNA#2 (gray bars). f.c., fold change. **(B)** IFN-α and IFN-γ, but not IFN-λ result in quickly ascending levels of nuclear IRF1 protein. Huh7.5 cells were treated with 5 ng/ml IFN-γ (top), 500 i.E./ml IFN-α (middle), or 50 ng/ml IFN-λ2 (bottom) for the displayed time points. Cells were lysed and cytoplasmatic and nuclear proteins were collected in separate fractions as described. The levels of IRF-1 were assessed *via* Western Blot, where Lamin B and γ-Adaptin are used as housekeeping proteins for the nuclear and cytosolic fractions, respectively. **(C)** *De novo* translation of IRF1 in response to type I IFN. Huh7.5 cells underwent pre-treatment with DMSO (top) or 50 µg/ml cycloheximide (bottom) for 6 h. After that, the cells were treated with 5 ng/ml IFNγ for the indicated time points. The amount of IRF-1 was analyzed *via* Immunoblot, where Lamin B and γ-Adaptin are used as housekeeping proteins for the nuclear and cytosolic fractions, respectively. In **(B, C)**, one out of three experiments (n=3) is shown as demonstration.

Given the convergence of type II and III IFN-signaling on intracellular Jak-Stat signaling, the lacking induction of IL-7 by IFN-λ2 warrants further explanation. As IRF-1 has been identified previously to serve as a transcription factor for IL-7 in epithelial cells (12), we first assessed the dependence of IFN-mediated IL-7 induction on IRF-1 in our system. As displayed in **Figure 2A**, suppression of IRF-1 in Huh-7.5 cells by siRNAs almost completely attenuated IFN-mediated induction of IL-7, confirming the relevance of this transcription factor for hepatocellular IL-7 production.

IRF-1-dependent gene expression requires influx of the IRF-1 protein into the nucleus. To better understand the mechanism of IL-7 induction by type I and II and not type III-IFN, we performed an analysis of IRF-1 protein levels in the cytosol and nucleus of Huh-7.5 cells after stimulation with these IFNs. Importantly, IRF-1 was undetectable in unstimulated Huh-7.5 cells, whereas treatment with IFN-α and IFN-γ, but not with IFN-λ2, resulted in a rapid and strong increase and nuclear influx of IRF-1 protein levels (**Figure 2B**). These results suggest that type I and II-, but not type III-IFNs are capable to induce the protein translation of IRF-1 and thereby restrict hepatic IL-7 production to these IFNs. To confirm induction of *de-novo* translation of the IRF-1 protein, IRF-1 protein levels in response to IFN-γ were subsequently assessed in Huh-7.5 cells with or without pre-treatment with cycloheximide, a specific inhibitor of protein translation. Indeed, pre-treatment with cycloheximide almost completely abrogated IFN-γ-mediated increase of IRF-1 protein levels (**Figure 2C**).

### Transcription of IRF-1 Amplifies Hepatocellular IL-7-Induction

**Figure 3:**
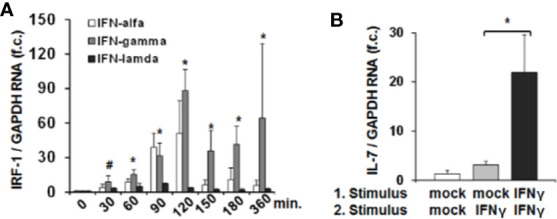
Interferon-response factor-1 (IRF1) transcription exaggerates interferon (IFN)-induced interleukin-7 (IL-7) expression. **(A)** Quantification of IRF-1 mRNA levels relative to housekeeping GAPDH mRNA in Huh-7.5 cells, which were treated for the indicated time points with IFN-α (500 i.E./ml), IFN-γ (5 ng/ml), or IFN-λ2 (50 ng/ml). IRF-1 mRNA levels relative to housekeeping GAPDH mRNA are expressed relative to untreated cells. Standard deviations of three experiments (n=3) performed with three replicates each are shown. *P < 0.05 and ^#^ *P*<0.1 for comparison of IFN-gamma treatment at the indicated time points versus baseline (i.e. mock), gray bars. Changes for IFN-alfa and IFN-lambda treatment are not significant. f.c., fold change. **(B)** Analysis of IL-7 mRNA levels relative to housekeeping GAPDH mRNA in Huh-7.5 cells, which were treated with PBS or one or two doses (baseline and after 6 h stimulation) of 5 ng/ml IFN-γ for 24 h. Standard deviations of two experiments (n=2) performed with three replicates each are shown. *P < 0.05 for indicated comparison. f.c., fold change.

Since IRF-1 is considered to be an ISG (i.e. gene expression (not translation) induced by IFN) (13) we next assessed whether type I, II and III IFNs induce IRF-1 mRNA expression in addition to the above described effects on protein translation. Stimulation of Huh-7.5 cells with IFN-α or IFN-γ resulted in a moderate or strong induction of IRF-1 mRNA, while IFN-λ2 had no relevant effect on IRF-1 transcription (**Figure 3A**). Hence, IFN-α and in particular IFN-γ, induce both early translation and in parallel transcription of IRF-1. These results suggest that *via* laying the foundation for further protein translation, IFN can amplify hepatocellular IRF-1 responses. Indeed, stimulation of Huh-7.5 cells with two subsequent doses of IFN-γ resulted in a significantly higher secretion of IL-7 compared to a single stimulus (**Figure 3B**). Hence, the IFN-induced cascade of IRF-1 mRNA induction and subsequent IRF-1 protein translation may represent a mechanism to overcome refractoriness of IRF-1 dependent ISG-expression.

### IL-7-Induced GSK3 Phosphorylation Augments the Lipopolysaccharide Response of Macrophages

**Figure 4:**
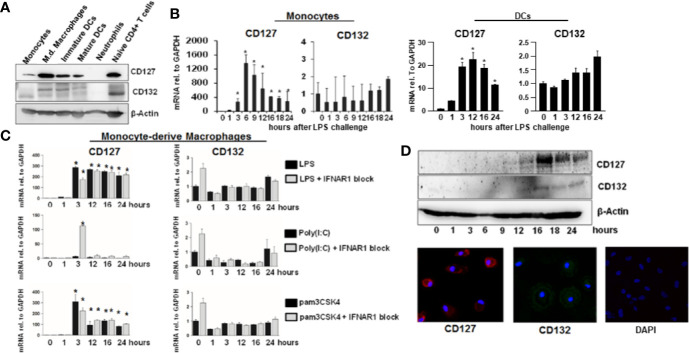
Inducible expression of CD127 in monocyte-derived macrophages. **(A)** Selected immune cells from the innate and adaptive immune system were isolated from Buffy Coats from healthy donors. Some cell types (Neutrophils, CD14^+^ monocytes, and naive CD4^+^ T cells were lysed directly after the isolation. Some CD14^+^ monocytes were matured into Monocyte-derived macrophages, immature and mature DCs as described in the methods section. The amount of CD127 and CD132 was analyzed *via* Western Blot with β-Actin as reference. One out of two experiments (n=2) is shown as demonstration. **(B)** mRNA levels of CD127 and CD132 relative to housekeeping GAPDH mRNA in freshly isolated monocytes and DCs, which were stimulated with 10 ng/ml lipopolysaccharide (LPS) for the displayed time points. Standard deviations of two experiments (n=2) performed with three replicates each are shown. *P < 0.05 for comparison of the indicated time points with baseline. **(C)** mRNA levels of CD127 and CD132 relative to housekeeping GAPDH mRNA in monocyte-derived macrophages, which were stimulated with 10 ng/ml LPS, 100 ng/ml poly(I:C) HMW or 100 ng/ml pams3CSK4, with or without 5 µg/ml antibody against IFNAR1, for the time points shown. Standard deviations of three experiments (n=3) performed with three replicates each are shown. *P < 0.05 for comparison of the indicated time points with baseline. **(D)** Monocyte-derived macrophages were stimulated with 10 ng/ml LPS for the displayed time points. The amount of CD127 and CD132 was assessed by Western Blot (top panel) and by immunofluorescence (bottom panel). One out of two experiments (n=2) is shown as demonstration.

To further explore possible functional implications of hepatic IL-7 expression, we assessed expression of the inducible IL-7 receptor in selected innate and adaptive immune cells relevant for liver pathology. As expected, naïve CD4+ T cells and dendritic cells (DCs) isolated from healthy volunteers expressed the interleukin-7 receptor-α CD127 (**Figure 4A**). Of note, CD127 was also detectable on monocytes and monocyte-derived macrophages (MDMs), a cell type which is not considered to be a classical target of IL-7 signaling, but CD127 expression in unstimulated monocytes and MDMs was highly variable (**Figure 4A**). However, as shown in **Figures 4B–D**, CD127 expression was consistently and strongly inducible by the Toll-like receptor (TLR)-4 agonist LPS in both monocytes and MDMs, suggesting a regulatory function of CD127 in these cells during LPS exposure. The inducibility was also detectable in DCs to a much lesser extent compared to monocytes and MDMs (**Figure 4B**). To assess, whether CD127 induction in macrophages is exclusive for TLR4-signaling, MDMs were stimulated with the TLR1/TLR2 agonist Pam3CSK4 or the TLR3 agonist poly I:C, respectively. As shown in **Figure 4C**, stimulation of MDMs with the TLR1/TLR2 agonist (mimicking bacterial infection) resulted in a strong increase of CD127 expression, comparable to that after LPS exposure, whereas no relevant induction of CD127 was observed after stimulation with the TLR3 agonist (mimicking viral infection). Induction of CD127 by TLR-agonists was independent from IFN-α/-β signaling, as evidenced by addition of a neutralizing antibody blocking the IFN-α receptor.

**Figure 5:**
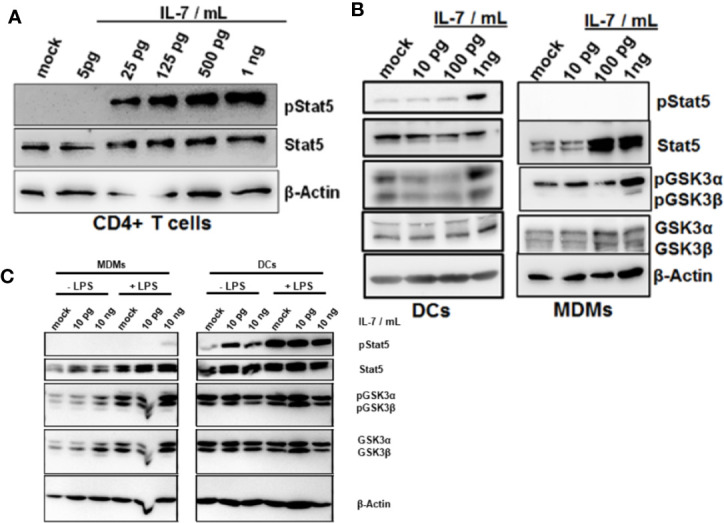
Phosphorylation of Glycogen synthase kinase-3 (GSK3) in dendritic cells (DCs) and macrophages in response to interleukin-7 (IL-7). **(A)** Naive CD4 T cells were treated with ascending concentrations of human IL-7 for 15 min. Presence of phosphorylated and total Stat5 was analyzed by Western Blot. **(B)** Human monocytes were differentiated into monocyte-derived DCs (left panel) or monocyte-derived macrophages (MDMs) (right panel). Cells were pre-incubated with 10 ng/ml lipopolysaccharide (LPS) for 48 h before treatment with the displayed concentrations of IL-7 for 15 min. Presence and phosphorylation status of proteins (i.e. Serine 21/9 phosphorylation on GSK3α and GSK3β respectively and Tyrosine 694 phosphorylation on Stat5) was assessed using Western Blot with β-Actin as control. **(C)** monocyte-derived macrophages (MDMs) and DCs were stimulated with or without 10 ng/ml LPS for 16 h before incubation with the different concentrations of IL-7 (10 pg/ml or 10 ng/ml) for 15 min. Presence and phosphorylation status of proteins (i.e. Serine 21/9 phosphorylation on GSK3α and GSK3β respectively and Tyrosine 694 phosphorylation on Stat5) was assessed using Western Blot with β-Actin as control. In **(A–C)**, one out of three experiments (n=3) is shown as demonstration.

Stat5 is the classical transcription factor mediating IL-7 signaling. As expected, naïve CD4+ T cells responded to IL-7 exposure by induction of Stat5 phosphorylation (**Figure 5A**). We next assessed whether IL-7 signaling is intact in innate immune cells as well. Importantly, both DCs and MDMs responded to IL-7 signaling much stronger after stimulation with LPS (**Figures 5B, C**). Furthermore, only DCs but not MDMs responded to IL-7 signaling by Stat5 phosphorylation (**Figure 5B**). In contrast, stimulation of both DCs and MDMs with IL-7 resulted in phosphorylation (i.e. inactivation) of Glycogen synthase kinase-3 (GSK3), another down-stream target of IL-7 signaling (**Figures 5B, C**). Whereas MDMs appeared to display stronger responses to IL-7 after LPS pretreatment this was not the case for the DCs (**Figure 5C**).

**Figure 6:**
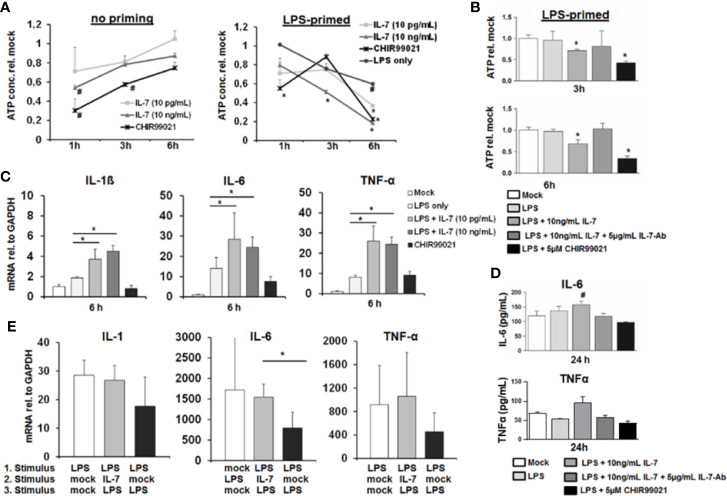
Interleukin-7 (IL-7) decreases intracellular ATP content of monocyte-derived macrophages but augments pro-inflammatory cytokine production in response to lipopolysaccharide (LPS). **(A)** Monocyte-derived macrophages (MDMs) underwent pre-incubation with or without 10 ng/ml LPS for 16 h before being stimulated with IL-7 or the Glycogen synthase kinase-3 (GSK3) Inhibitor CHIR99021 (5 µM) for the displayed time points. Then the intracellular ATP content relative to unstimulated controls was analyzed. Standard deviations of three experiments (n=3) performed with three replicates each are shown. *P < 0.05 and ^#^ *P* < 0.1 for comparison of the indicated condition at the indicated time point versus mock. **(B)** MDMs were pre-incubated with 10 ng/ml LPS for 16 h prior to stimulation with 10 ng/ml IL-7 in presence or absence of 5 µg/ml IL-7 antibody or the GSK3 Inhibitor CHIR99021 (5 µM) for the time points shown. Then the intracellular ATP content relative to unstimulated controls was analyzed by luciferase reaction. Standard deviations of three experiments (n=3) performed with three replicates each are shown. *P < 0.05 for comparison of the indicated condition versus mock. **(C)** Analysis of mRNA levels of IL-1β, IL-6, and tumor necrosis factor-α (TNF-α) relative to housekeeping GAPDH mRNA in MDMs pre-stimulated with 10 ng/ml LPS as indicated, which were then treated with the indicated reagents for 6 h. Standard deviations of three experiments (n=3) performed with three replicates each are shown. *P < 0.05 for indicated comparisons. **(D)** MDMs underwent pre-incubation with 10 ng/ml LPS for 16 h prior to stimulation with 10 ng/ml IL-7 in presence or absence of 5 µg/ml IL-7 antibody or the GSK3 Inhibitor CHIR99021 (5 µM) for 24 h. The amount of IL-6 (top) and TNFα (bottom) in cell culture supernatant was quantified by ELISA. Standard deviations of three experiments (n=3) performed with three replicates each are shown. ^#^ *P* < 0.1 for comparison of the indicated condition versus mock. **(E)** Assessment of mRNA levels of IL-1β, IL-6, and TNFα relative to housekeeping GAPDH mRNA in MDMs pre-incubated with or without two sequential stimuli with 10 ng/ml LPS as shown, and which were co-stimulated with or without 10 ng/ml IL-7 30 min after the first LPS stimulus. Standard deviations of three experiments (n=3) performed with three replicates each are shown. *P < 0.05 for indicated comparisons.

GSK3 is a key kinase involved in cellular energy metabolism and survival (14). Furthermore, activation of GSK3 has been shown to mediate tolerance to LPS of macrophages in response to TNF-α (15). We therefore tested the impact of IL-7 signaling on ATP stocks, survival and cytokine secretion of MDMs. As shown in **Figure 6A**, IL-7 signaling decreased intracellular ATP levels in MDMs within hours, to an extend comparable to a small molecule inhibitor of GSK3. In non-primed MDMs the changes appear to be the strongest at the shortest timepoints. In line with the above described induction of GSK3 phosphorylation, the decrease of intracellular ATP in MDMs appeared to be stronger and was longer lasting in cells pretreated with LPS (**Figure 6A**). The decrease of intracellular ATP was specific to IL-7 signaling, as evidenced by amelioration of the ATP decrease in the presence of a neutralizing antibody against the IL-7 receptor (**Figure 6B**). Furthermore, a comparable decrease of intracellular ATP concentrations was observed after stimulation of MDMs with IL-7 after pre-treatment with the TLR1/2 agonist Pam3CSK4 (**SI Figure 2A**).

Next, we assessed the impact of IL-7 signaling on LPS-induced production of pro-inflammatory cytokines in MDMs. Stimulation of LPS-primed MDMs with IL-7 resulted in a significantly higher induction of the pro-inflammatory cytokines IL-1β, IL-6, and TNF-α mRNA levels (**Figure 6C**), and – as shown exemplarily for IL-6 and TNFα - secretion of this cytokine to cell culture supernatants (**Figure 6D**). Again, augmentation of pro-inflammatory cytokine gene expression in the presence of IL-7 was specific for IL-7 receptor signaling, as shown by the addition of a neutralizing antibody against IL-7 (**SI Figure 3**). Furthermore, co-stimulation of MDMs with LPS and IL-7 blunted tolerance to a second LPS stimulus compared to two stimuli with LPS in the absence of IL-7 (**Figure 6E**). This phenomenon was also observed in a co-culture experiment of LPS-stimulated MDMs with type I-IFN-primed Huh-7.5 cells, in which the addition of a neutralizing antibody against IL-7 (partially) decreased LPS-mediated IL-6 induction and (partially) increased ATP concentrations in MDMs (**SI Figure 3**). Finally, a moderate augmentation of pro-inflammatory cytokine gene expression by IL-7-stimulation was observed in MDMs stimulated with the TLR1/2 agonist Pam3CSK4 (**SI Figure 3**).

**Figure 7:**
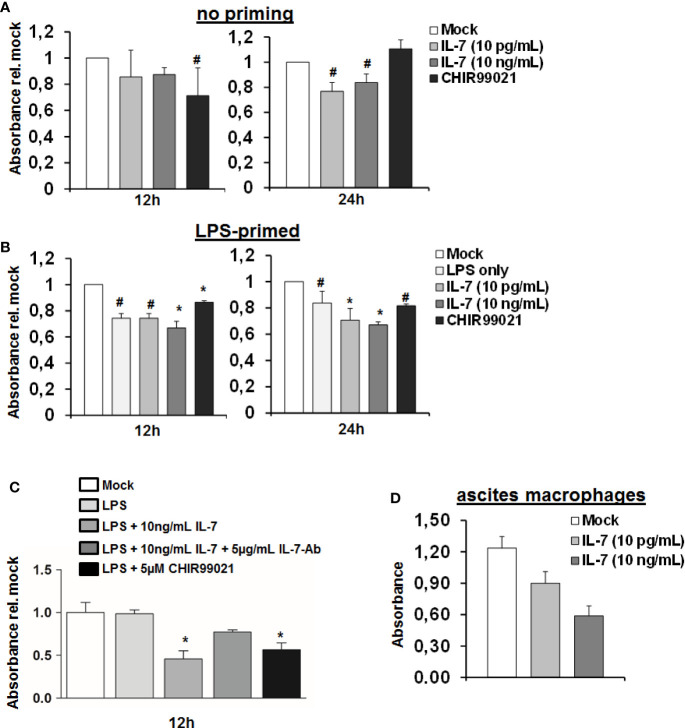
Interleukin-7 (IL-7) decreases monocyte-derived macrophage viability. **(A, B)** Human monocyte-derived macrophages (MDMs) were pre-stimulated with **(B)** or without **(A)** 10 ng/ml lipopolysaccharide (LPS) for 16 h. Subsequently, cells were stimulated with the displayed concentrations of IL-7 or the Glycogen synthase kinase-3 (GSK3) inhibitor CHIR99021. Cellular viability was estimated using the WST-1 reagent at an absorbance of 450 nm relative to unstimulated cells. Standard deviations of three experiments (n=3) performed with three replicates each are shown. *P < 0.05 and ^#^ *P* < 0.1 for comparison of the indicated condition at the indicated time point versus mock. **(C)** MDMs were stimulated as in B) with or without addition of a neutralizing antibody against IL-7. *P < 0.05 for comparison of the indicated condition versus mock. Standard deviations of two experiments (n=2) performed with three replicates each are shown. **(D)** Macrophages were isolated from ascites fluid of two liver cirrhosis patients (n=2, 3 replicates each) with spontaneous-bacterial peritonitis (SBP) and directly stimulated with IL-7 at the displayed doses. Cell viability using WST-1 reagent was analyzed after 24 h.

The above indicated experiments show that IL-7 signaling increases production of LPS-induced pro-inflammatory cytokine production and ameliorates LPS tolerance in macrophages, which is, however, accompanied by reduced intracellular ATP concentrations. We therefore assessed cellular viability of MDMs stimulated with LPS and IL-7 using the WST-1 reagent. As shown in **Figure 7**, IL-7 signaling appeared to be on the cost of a reduced viability/cellular integrity of MDMs (in particular after priming with LPS). Yet, the impact of IL-7 on cellular viability was only partially restored by the addition of a neutralizing antibody against IL-7 (**Figure 7C**). A negative impact of IL-7 on cellular viability was also observed in peritoneal macrophages derived from two patients with spontaneous bacterial peritonitis (**Figure 7D**). Collectively, these data suggest that IL-7 may enhance innate, macrophage-mediated immunity *via* inhibition of GSK3 on the cost of fitness of these cells, contrasting its well-known Stat5-mediated pro-survival effect, which IL-7 displays on T cells (14).

### The IFN-IL-7-GSK3 Circuit Is Present in Patients With Liver Cirrhosis

**Figure 8:**
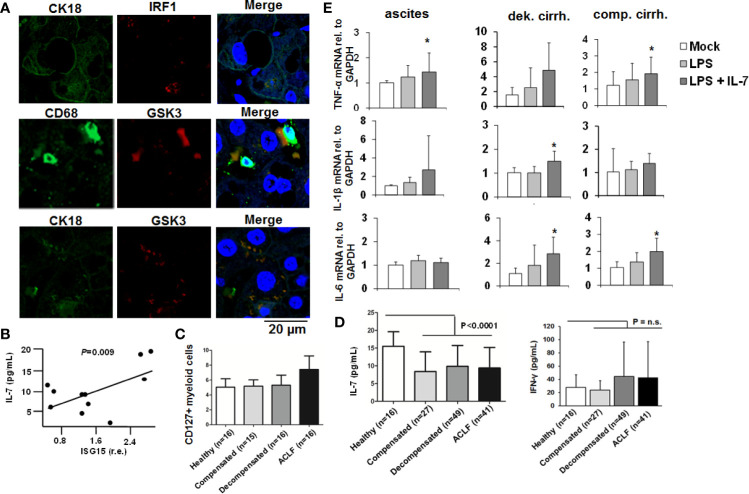
The IFN-IRF-1-IL-7 axis is present in patients with liver cirrhosis. **(A)** Presence of the proteins interferon-response factor-1 (IRF1), Glycogen synthase kinase-3 (GSK3), CD68 as a macrophage marker, and CK18 as part of the hepatocellular cytoskeleton was analyzed by immunofluorescence in paraffin-embedded tissue of a representative patient with liver cirrhosis. One out of two experiments (n=2) is shown as demonstration. **(B)** Interferon-stimulated gene (ISG)-15 relative to GAPDH mRNA concentration was quantified by quantitative PCR in (PBMCs of 11 randomly selected patients with liver cirrhosis from **(C)** and correlated with serum concentrations of interleukin-7 (IL-7). *P* value for Pearson correlation. **(C)** Flow cytometric analyses of the percentage of CD127 positive myeloid cells relative to all myeloid cells from patients with liver cirrhosis and available PBMCs or healthy controls. **(D)** Serum concentrations of IL-7 and interferon-(IFN)-γ in healthy controls and in patients with different liver cirrhosis stages or with acute-on-chronic liver failure (ACLF) were determined by ELISA. *P* for comparison between healthy controls and all patients with liver cirrhosis. **(E)** Monocytes from peripheral blood and ascites-derived macrophages of patients with ascites (n=4), compensated liver cirrhosis (n=4) and decompensated liver cirrhosis (n=4, all measured in duplicate) were stimulated *ex vivo* with 10 ng/ml IL-7 and/or 10 ng/ml LPS, as indicated, for 6 h. Then, quantitative analysis of mRNA of IL-1β, IL-6, and tumor necrosis factor α (TNFα) levels relative to housekeeping GAPDH mRNA was performed. *P < 0.05 for comparison of the indicated condition versus mock; other comparisons were not significant.

To further explore the *in vivo* relevance of our findings, we assessed IRF-1 and GSK3-expression in liver specimens of patients with liver cirrhosis. As shown in **Figure 8A**, IRF-1 was detectable in hepatocytes of patients with liver cirrhosis, whereas GSK-3 co-localized not only with markers of hepatocytes, but also with markers of liver macrophages. Furthermore, ISG-15 expression in PBMCs of patients with liver cirrhosis correlated significantly with IL-7-serum levels (**Figure 8B**), suggesting an *in vivo* correlation between the activity of the IFN-system and IL-7 production. In addition, myeloid cells from patients with ACLF expressed higher levels of the LPS-inducible IL-7 receptor CD127 compared to patients with liver cirrhosis and to ACLF or healthy controls (**Figure 8C**). However, a systematic analyses of IL-7 serum levels in patients with liver cirrhosis and healthy individuals revealed significantly lower IL-7 serum concentrations in patients with liver cirrhosis including patients with ACLF compared to healthy controls, despite of high IFN-γ serum concentrations in patients with decompensated liver cirrhosis and ACLF [**Figure 8D**, detailed patient characteristics are described in (9)]. Hence, IL-7 deficiency in patients with liver cirrhosis could be a factor of the well-known LPS tolerance and defective adaptive immunity in these patients (16). We therefore assessed whether IL-7 signaling can ameliorate LPS-tolerance in blood-derived monocytes and ascites-derived macrophages from patients with liver cirrhosis *ex vivo*. As shown in **Figure 8E**, the addition of IL-7 numerically increased LPS-induced gene expression of pro-inflammatory cytokines in these cells, although there were considerable variations between monocytes/macrophages from different patients.

## Discussion

IL-7 is a non-redundant key player required for important adaptive immune cells to proliferate and to be maintained. In a multiple sclerosis animal model, hepatocellular IL-7, which was induced by toll-like receptor (TLR) signaling, was described as an important source of IL-7, which enables survival of CD8+ and CD4+ T cells (4). In the present study we identify IRF-1 as a transcription factor restricting hepatic IL-7-induction to type I and II IFN. In addition, we show that LPS-primed macrophages respond to IL-7 signaling by distinct downstream signaling pathways compared to CD4+ T cells, i.e. by phosphorylation and inactivation of macrophage GSK3. IL-7-induced GSK3 inactivation augments the response of macrophages to LPS and blunts LPS tolerance. Finally, we show that patients with liver cirrhosis have reduced concentrations of IL-7 in serum in comparison to healthy controls, a factor which may contribute to LPS tolerance, a cardinal feature of liver cirrhosis-associated immunodysfunction.

All types of Interferons are central proteins of early hepatic innate immunity. Infections with bacteria or viruses as well as translocation of bacterial components like Lipopolysaccharide from the intestine can induce all IFN types in hepatocytes (type I and III IFN), or in liver sinusoidal endothelial cells, in innate immune cells like macrophages (type I and II IFN) *via* TLR signaling (5, 17, 18). IFN signaling pathways induce a variety of ISGs. Those ISGs upregulated by type I and III IFNs display a remarkable overlap because the build-up of heterodimers consisting of pStat1-pStat2 is thought to be the main event downstream of both their receptors (5, 17). On the other hand, signaling of IFN-γ mostly leads to the presence of pStat1-pStat1 homodimers, and as a consequence, ISGs expressed after IFN-γ stimulation are partially different from those resulting of type I and III IFN treatment (19). In the present study we describe an unconventional mechanism of inducing ISG, which is *de novo* translation and transcription of IRF-1 protein/mRNA simultaneously in response to IFN-α and IFN-γ, but not IFN-λ. IRF-1 transcription/translation therefore seems to limit expression of a class of ISGs like IL-7 to the on the other hand rather distinct type I and II IFN system. Furthermore, parallel induction of IRF-1 mRNA transcription and protein translation serves as a mechanistic approach of longer-lasting ISG-expression and therefore might be a possible modality to circumvent IFN-refractoriness for distinct ISGs (see **Figure 3**).

The finding that IFN-λ does not induce IRF-1 transcription/translation and the following hepatocellular IL-7 expression is potentially important for clinical aspects. IFN-λ as a type III IFN undergoes clinical evaluation for treating chronic hepatitis B (20). The key advantage of a new IFN-λ therapy compared to therapies with pegylated IFN-α is its better tolerability, because only distinct cell types such as hepatocytes and specific epithelial cells express the receptors for IFN-λ. However, establishing efficient adaptive immune responses is crucial to clear the hepatitis B virus, and as IFN-λ failed to induce hepatocellular IL-7 that might cause less efficient T cell immune responses compared to conventional therapies with pegylated IFN-α. Beyond their important antiviral features, IFNs show essential anti-proliferative and anti-tumor properties (21). IRF-1 is a pivotal suppressor of distinct malignancies (22). In view of our findings, it may therefore be speculated that IFN-λ is a less potent anti-tumor agent compared to IFN-α or IFN-γ.

Another finding of our study is that not only hepatocytes, but also LSECs are capable to produce IL-7 in response to IFN. LSECs are important components of the hepatic immune system and the front cell barrier to pathogens or immune cells from the circulation which enter the liver. Therefore, IL-7 production by LSECs provides signals to lymphocytes from the circulation or macrophages recruited to the liver (23). While it is known, that CD8+ T cells, CD4+ cells, or mucosal associated invariant T cells (MAIT) highly depend on IL-7 and the resulting Jak-Stat5 signaling, only few reports have mentioned an importance for IL-7 signaling in innate immune cells such as macrophages. For example, expression of the IL-7 receptor has been shown on macrophages in humans with rheumatoid arthritis (24). IL-7 resulted in production of pro-angiogenic factors by macrophages, but the IL-7 signaling cascade in macrophages has not been investigated in this report. Our finding that IL-7 predominantly promotes inhibitory phosphorylation of GSK3, instead of Stat5, in macrophages provides additional knowledge to understand the role of IL-7 in macrophage immunoregulation.

In the present study, IL-7 mediated inhibition of GSK3 in Lipopolysaccharide-primed macrophages resulted in elevated cytokine secretion and reduced Lipopolysaccharide tolerance of macrophages. These data are in line with a former study displaying that TNF-α-induced activation of GSK3 (in contrast to IL-7 mediated GSK3-inhibition) results in endotoxin tolerance and survival in macrophages (15), as well as with a previous report that has identified a role of GSK3 in LPS-induced inflammation in patients with decompensated liver cirrhosis (25). However, the role of GSK3 in cellular pathways leading to cell survival or cytokine secretion is controversial in literature as another study marked an inactive GSK3 as an important mediator of elevated IL10 production in human monocytes (26). The authors inhibited GSK3 first and then pulsed the monocytes afterwards with different TLR agonists to compare cytokine secretion. Yet, we performed this study mainly in monocyte-derived macrophages, which may undergo changes in signaling pathways due to the 6day incubation period with the growth factor M-CSF. In addition, for our cells the TLR agonist was the pre-stimulus followed by the IL-7, which increased inhibitory phosphorylations on GSK3, as we intended to mimic the hepatic situation where all cell types face continuous TLR agonist supply by the portal vein. From a clinical point of view, our data suggest that IL-7 (or inhibition GSK3 in macrophages) might be of value to prevent and treat infections in liver cirrhosis patients and thereby to prevent and ameliorate the development of ACLF and death. This notion is further supported by our result that liver cirrhosis patients including patients with ACLF display lower concentrations of IL-7 in serum compared to healthy controls, a scenario in which therapeutic application of IL-7 could be particularly relevant. Indeed, we could observe a trend of an IL-7-mediated blunted LPS tolerance in monocytes and ascites macrophages of patients with liver cirrhosis *ex vivo*. Yet, the considerable variation between different patients requires further studies including larger patient numbers to draw definite conclusions and to understand possible (genetic and non-genetic) confounders of this phenomenon. Of note, the use of IL-7 as therapy in bacterial and viral infections is under investigation (27, 28). Although we can only speculate about the mechanism of reduced IL-7 serum concentrations in patients with liver cirrhosis, an explanation could be the significantly reduced liver mass of these patients with the consequence of a lower hepatic cell pool being able to secrete IL-7 in response to IFN.

The observed decrease of intracellular ATP stocks and reduced viability of MDMs in response to IL-7 suggests that the augmentation of pro-inflammatory cytokine production by macrophages goes along with lower fitness in these cells. Yet, we must acknowledge that the functional mechanism of reduced ATP stocks in MDMs remains to be elucidated. It is possible, that this finding simply reflects an increased consumption of energy due to increased production of cytokines and other mediators. Another possibility would be a direct influence of GSK3 inhibition on macrophage metabolism, a hypothesis which might be investigated in future studies. In this regard, it may also be interesting to assess whether IL-7-induced regulation of GSK3 may promote a switch from quiescent (long-living) to effector macrophages, perhaps *via* modulating macrophage metabolism or epigenetic signatures.

In conclusion, our present study suggests the presence of a pro-inflammatory signaling cascade in where type I and II IFNs induce hepatocellular Interleukin-7 in an IRF-1-dependent manner. Beyond its role in the establishment and maintenance of adaptive immune responses, IL-7 appears to augment the response of macrophages to LPS and to ameliorate LPS tolerance, which may improve innate immune responses against invading pathogens.

## Data Availability Statement

The original contributions presented in the study are included in the article/**Supplementary Material** further inquiries can be directed to the corresponding author.

## Ethics Statement

The studies involving human participants were reviewed and approved by the Ethics committee University Hospital Frankfurt. The patients/participants provided their written informed consent to participate in this study.

## Author Contributions

The authors have contributed to the manuscript by planning the study (SR and CML), collecting the data (SR, CC, MS, KS, CW, and CML), analyzing and interpreting the data (SR, CC, KS, UD, SZ, CW, and CML), and preparing and revising the manuscript (all authors). All authors contributed to the article and approved the submitted version.

## Funding

CML is supported by the Deutsche Forschungsgemeinschaft (LA 2806/2-1 and LA 2806/5-1 to CML) and by the IFORES Förderprogramm of the University Duisburg-Essen.

## Conflict of Interest

The authors declare that the research was conducted in the absence of any commercial or financial relationships that could be construed as a potential conflict of interest.
