# Supplementary material for: Translation of IRF-1 Restricts Hepatic Interleukin-7 Production to Types I and II Interferons: Implications for Hepatic Immunity
Source: Front Immunol. 2021 Jan 14;11:581352. doi: 10.3389/fimmu.2020.581352 (PMC7874116; doi:10.3389/fimmu.2020.581352)
Supplement: Supplementary file 1 [file DataSheet_1.docx]

**Supplementary Information Rueschenbaum et al.**

**Supplementary figures**


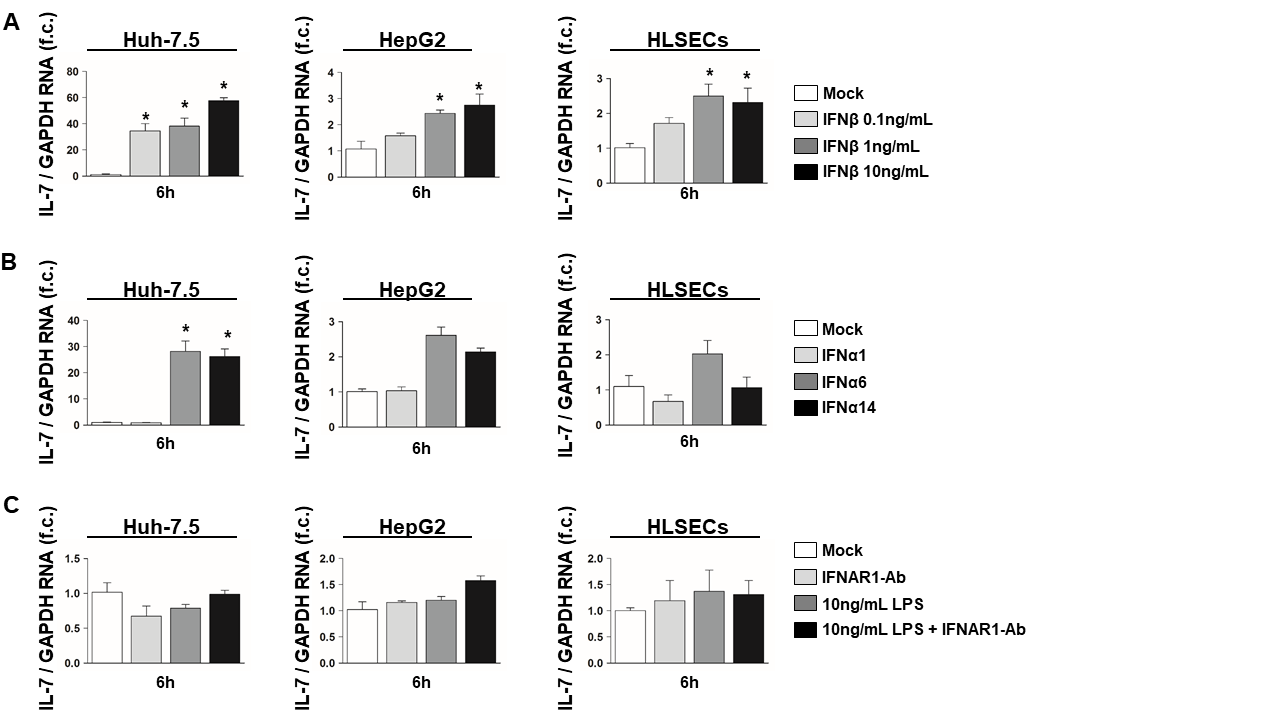


**SI Figure 1. Induction of IL-7 by IFNβ and distinct IFNα subtypes. A)** Quantification of IL-7 mRNA levels relative to GAPDH mRNA in Huh-7.5 cells (left), HepG2 cells (middle) and human liver sinusoidal endothelial cells (HLSECs) (right), which were stimulated for 6 hours with IFN-β at the indicated dosages. IL-7 expression is shown relative to untreated cells. Standard deviations of 3 independent experiments (n=3) performed with 3 replicates each are shown. **P* < 0.05 for comparison of the indicated condition with mock. f.c., fold change. **B)** Quantification of IL-7 mRNA levels relative to GAPDH mRNA in Huh-7.5 cells (left), HepG2 cells (middle) and HLSECs (right), which were stimulated for 6 hours with 500 IU/ml of the indicated IFN-α subtypes. IL-7 expression is shown relative to untreated cells. Standard deviations of 3 independent experiments (n=3) performed with 3 replicates each are shown. **P* < 0.05 for comparison of the indicated condition with mock. f.c., fold change. IU, international units. **C)** No induction of hepatic IL-7 by LPS. Quantification of IL-7 mRNA levels relative to GAPDH mRNA in Huh-7.5 cells (left), HepG2 cells (middle) and HLSECs (right), which were treated for 6 hours with 5µg/mL IFNAR1 antibody, 10 ng/mL LPS, or both. IL-7 expression is shown relative to untreated cells. Standard deviations of 3 independent experiments (n=3) performed with 3 replicates each are shown. f.c., fold change.

**SI Figure 2. IL-7 decreases intracellular ATP content of monocyte-derived macrophages but augments pro-inflammatory cytokine production in response to TLR1/TLR2 agonist.** **A)** MDMs were primed with 100ng/ml pam3CSK4 for 16 hours prior to stimulation with 10ng/ml IL-7 in presence or absence of 5µg/ml neutralizing IL-7 antibody or the GSK3 Inhibitor CHIR99021 (5µM) for the indicated time points. Cells were lysed and intracellular ATP content was measured by luciferase reaction. Standard deviations of 3 experiments (n=3) performed with 3 replicates each are shown. **P* < 0.05 for comparisons of the indicated condition versus mock. **B)** Quantification of IL-1β, IL-6 and TNFα mRNA levels relative to GAPDH mRNA in MDMs primed with 100ng/ml LPS as indicated, which were then stimulated with the indicated reagents for 6 hours. Standard deviations of 3 experiments (n=3) performed with 3 replicates each are shown. **P* < 0.05 for comparisons of the indicated condition versus mock.

**SI Figure 3. Co-culture model. A**) Proof-of-principle that binding of IL-7 by a neutralizing antibody reduces IL-7-augmented cytokine secretion by monocyte-derived macrophages. Quantification of IL-1β, IL-6 and TNFα mRNA levels relative to GAPDH mRNA was performed in MDMs primed with 10ng/ml LPS as indicated, which were then stimulated with 10ng/ml IL-7 in the presence or absence of 5µg/ml neutralizing antibody against IL-7 or 5µM CHIR99021 for 6 hours. Standard deviations of 3 experiments (n=3) performed with 3 replicates each are shown. **P* < 0.05 for comparison of the indicated condition vs. mock or for the comparison indicated by the bar. **B)** Scheme of co-culture experiment setup. **C)** After 6 hours in co-culture with the indicated conditions, macrophages were lysed and intracellular ATP content was measured by luciferase reaction. Standard deviations of 3 experiments (n=3) performed with 3 replicates each are shown. **P* < 0.05 for comparison of the indicated condition vs. mock or for the comparison indicated by the bar. **D)** After 6 hours in co-culture with the indicated conditions, macrophages were lysed and IL-6 mRNA was determined relative to GAPDH. Standard deviations of 2 experiments (n=2) performed with 3 replicates each are shown. **P* < 0.05 for comparison of the indicated condition vs. mock or for the comparison indicated by the bar. D) After 24 hours in co-culture with the indicated conditions, cell viability of Huh-7.5 was assessed using WST-1 reagent. Standard deviations of 2 experiments (n=2) performed with 3 replicates each are shown. **P* < 0.05 for comparison of the indicated condition vs. mock.
